# Abdominal apoplexy because of the rupture of gastroduodenal artery and inferior pancreaticoduodenal artery: A case report

**DOI:** 10.1097/MD.0000000000008264

**Published:** 2017-10-27

**Authors:** Hangyan Wang, Dianrong Xiu

**Affiliations:** Department of General Surgery, Peking University Third Hospital, Beijing, P.R. China.

**Keywords:** abdominal apoplexy, embolization, hemorrhage

## Abstract

**Rationale:**

Abdominal apoplexy is a rare and fatal emergency event, which is coined as a comparison to the cerebrovascular apoplexy. The exact mechanism of abdominal apoplexy was unclear, but arteriosclerosis, hypertension, abdominal aneurysm, and other predisposing angiopathy were considered to be the main reasons of abdominal apoplexy. The development of the imaging technology gave us more opportunities to confirm the diagnosis of abdominal apoplexy. However, the diagnosis and identification of the bleeding sites still continued to be a challenge.

**Patient concerns:**

A 55-year-old man presented to the emergency department with chief complains of sudden severe abdominal pain.

**Diagnosis:**

The patient was diagnosed as abdominal apoplexy with 2 synchronous bleeding sites.

**Interventions:**

Angiography confirmed diagnosis of abdominal apoplexy and revealed 2 synchronous bleeding sites in gastroduodenal artery (GDA) and inferior pancreaticoduodenal artery (IPDA). Transcatheter embolization was performed immediately.

**Outcomes:**

The patient recovered and was discharged very soon. Two months later, the patient totally recovered and the hematoma disappeared in the CT imaging.

**Lessons:**

The reported case is rare, given the very low incidence of abdominal apoplexy with 2 synchronous bleeding sites in GDA and IPDA. The awareness of abdominal apoplexy was still the key point in the management of this disease. Quick diagnosis by the imaging and immediate embolization were very important for the treatment.

## 1 Introduction

Abdominal apoplexy is a rare and fatal emergency event, which was replaced by “idiopathic spontaneous intraperitoneal hemorrhage (ISIH).” The term abdominal apoplexy is still widely used, because it is coined as a comparison to the cerebrovascular apoplexy. Although not completely understood, there may be the similar feature and etiology between abdominal apoplexy and cerebrovascular apoplexy. Historically, the diagnosis of abdominal apoplexy and the site of bleeding could only be confirmed during the surgical intervention or autopsy, and eventually around 30% of cases had no identifiable source.^[1]^ But the development of the imaging technology gave us more opportunities to suspect or confirm the diagnosis of abdominal apoplexy. However, the diagnosis continued to be delayed because of rarity of the process and lack of suspicion.^[2]^ The preoperative diagnosis remains challenging, despite the recent radiologic advances and surgical exploration is often unavoidable for the diagnosis and treatment.^[3]^

Usually the abdominal apoplexy had only 1 identified bleeding site, but we reported a case presenting with 2 synchronous bleeding sites, involving gastroduodenal artery (GDA) and inferior pancreaticoduodenal artery (IPDA).

## 2 Case report

**Figure 1:**
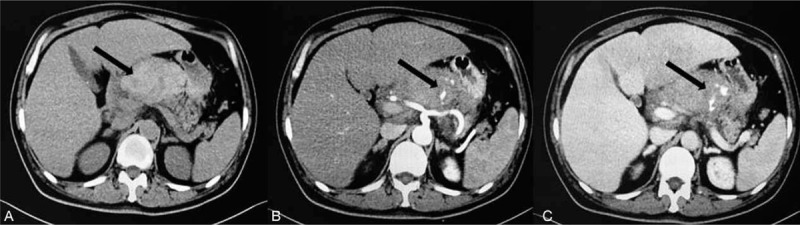
Enhanced CT scans of the patient before intervention. (A) Black arrow shows a 12 cm hematoma near the head of pancreas and duodenum. (B) Artery phase, black arrow shows extravasation of the contrast medium. (C) Vein phase, black arrow shows the enlargement of extravasation of the contrast medium. CT = computed tomography.

**Figure 2:**
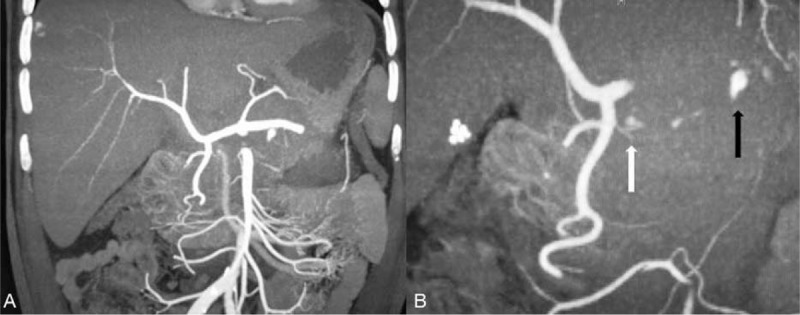
Enhanced CT scans (coronal view) before intervention. (A) The course of superior mesentery artery and celiac trunk. (B) Black arrow shows extravasation from IPDA and white arrow shows extravasation from GDA. CT = computed tomography, GDA = gastroduodenal artery, IPDA = inferior pancreaticoduodenal artery.

A 55-year-old man presented to the emergency department (ED) with chief complains of abdominal pain. He suffered sudden upper abdominal pain with nausea and vomiting. The pain was severe and the patient had to keep a curled up posture to relieve the pain. He had progression of his pain through to the back and subjective sensation of abdominal distention. His past medical history included hypertension, hyperlipidemia, and cerebral infraction. He suffered ischemic cerebral infraction 4 years ago and recovered without limb dyskinesia. He had no other antecedent history. He denied use of tobacco or alcohol. There were no medication changes recently. Review of systems was generally unremarkable, and he specifically denied stool changes or other associations. He denied any trauma. Vital signs were remarkable for a blood pressure of 116/70 mm Hg with a heart rate of 92 beats/min. He had upper abdominal tenderness with absence of bowel sounds. His blood pressure remained stable. Hemoglobin was 10.2 g/dL with a hematocrit of 37.5%. Serum amylase was elevated at 150 U/L (normal 30–110 U/L). Serum lipase was elevated at 696 (normal 23–300 U/L). The other chemistry studies were essentially normal. Coagulation studies were all within the normal range. Due to the patient's body habitus and bowel gas, there were no positive signs in sonography. Considering the feature of abdominal pain with radiation to the back and the history of cerebrovascular apoplexy, the visceral vascular disease was highly suspected. Because the patient remained hemodynamically stable, he was sent for an abdominal enhanced computed tomography (CT) scan. The enhanced CT scan was negative for abdominal aneurysm, but showed a 12 cm mass with extravasation of the contrast medium (Figs. 1 and 2). There was no fluid in the peritoneal cavity. The results of this scan showed retroperitoneal hematoma around the head of pancreas and duodenum. Then the patient accepted angiography, which revealed synchronous bleeding of GDA and IPDA.

**Figure 3:**
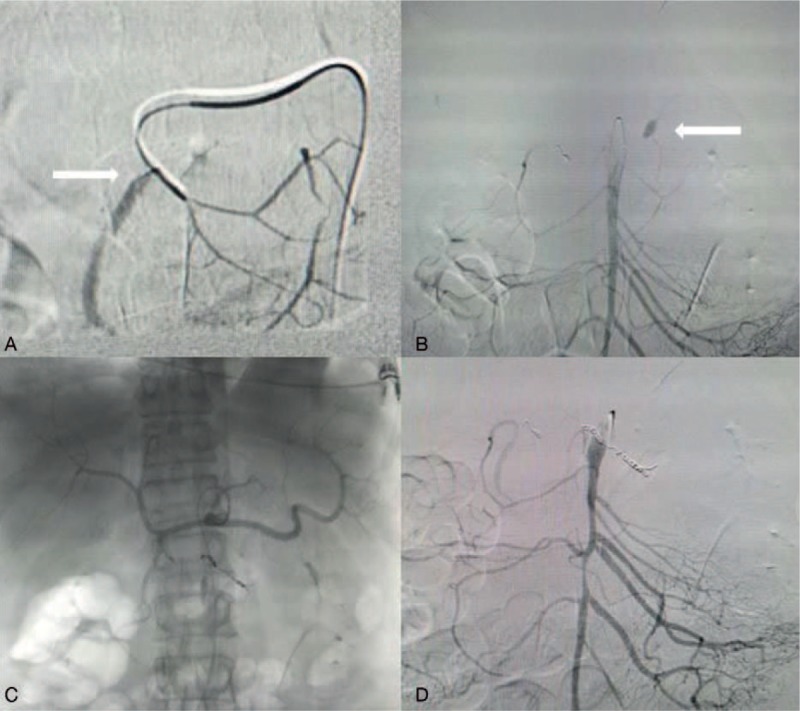
Angiography and transcatheter embolization. (A) White arrow shows extravasation from the GDA. (B) White arrow shows extravasation from IPDA. (C, D) Transcatheter embolization of the branches of GDA and IPDA. GDA = gastroduodenal artery, IPDA = inferior pancreaticoduodenal artery.

**Figure 4:**
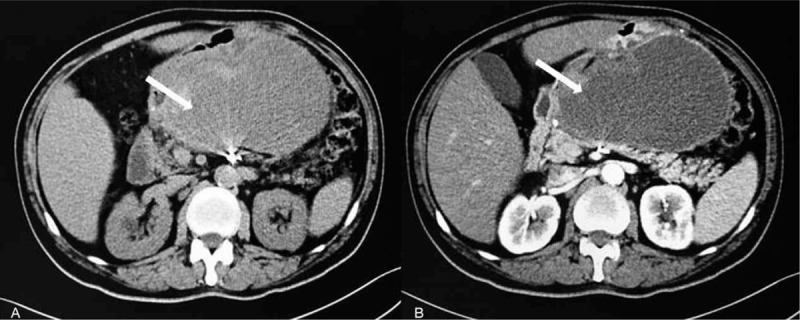
Enhanced CT scans 1 mo after intervention. (A) Non-contrast-enhanced phase. (B) Artery phase. White arrow shows the hematoma between the stomach and pancreas. CT = computed tomography.

**Figure 5:**
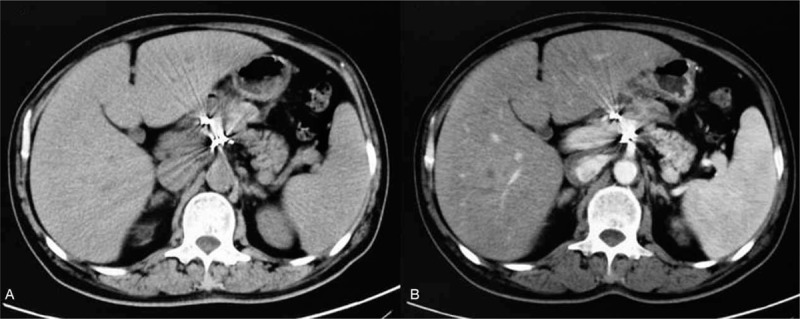
Enhanced CT scans 2 mo after intervention. (A) Non-contrast-enhanced phase. (B) Artery phase. The hematoma significantly diminishes in the imaging. CT = computed tomography.

Transcatheter embolization was performed to stop bleeding (Fig. 3). After the embolization the pain significantly relieved with stable hemodynamics and hemoglobin level. The patients discharged 5 days later. One month later, the patient recovered without any symptoms except the hematoma in the CT imaging (Fig. 4). Two month later, the patient totally recovered and the hematoma disappeared in the CT imaging (Fig. 5).

The patient signed informed consents. In our case the patient accepted regular and proved therapy in the ED, so the ethical approval was not necessary.

## 3 Discussion

Abdominal apoplexy defined the condition of intraperitoneal or retroperitoneal hemorrhage. Apoplexy is derived from the Greek word “apoplessein” which means sudden paralysis from the breaking or obstruction of vessels in the brain. The catastrophic nature and spontaneity of this disease makes in an appropriate abdominal equivalent to cerebrovascular apoplexy.^[4]^ The definition of abdominal apoplexy or ISIH has been refined to encompass nontraumatic, spontaneous hemorrhage due to rupture of the smaller abdominal arteries or veins, and the definition excludes hemorrhage from aortic aneurysm or dissection, gynecological lesions, ectopic pregnancy, or visceral malignancy.^[5]^ Abdominal apoplexy has a low reported incidence. Kennedy reported 50 cases of abdominal apoplexy between 1909 and 1965.^[6]^ Carter reviewed a series of 85 cases in 1966 and Kleinsasser reviewed a series of 83 cases in 1970.^[7,8]^ Carmeci reported only 110 cases between 1909 and 1998.^[9]^

The exact mechanism of abdominal apoplexy was unclear but it was likely due to the weakness of the tunica media in the small vessels with angiopathy such as aneurysms or atherosclerosis, which predisposes vessel rupture in the face of abrupt increase in blood pressure. Pathology specimens also regularly demonstrate disruption of elastic laminae.^[10]^

Historically, abdominal aneurysm was considered to be the main reason of abdominal apoplexy. Of all visceral artery aneurysms, 60% involve the splenic artery, 20% hepatic artery, 5% superior mesenteric artery, and 3% gastric artery and gastroepiploic arteries.^[11]^ However, the splenic artery only account for about 10% of abdominal apoplexy cases with identifiable source of bleeding and the hepatic artery was extremely rarely involved. The most popular bleeding sites of abdominal apoplexy were left gastric artery, superior mesenteric artery, and middle colic artery.^[7,8]^ The mismatching between the distribution of visceral artery aneurysms and the bleeding sites of abdominal apoplexy might give us some insight into the mechanism of abdominal apoplexy. An aneurysmic stage did not necessarily precede the spontaneous rupture of a visceral artery^[12]^ and bleeding was frequently found in conjunction with hypertension (33–50%) and atherosclerosis (80–87%).^[1]^ So besides visceral artery aneurysms, the hypertension and atherosclerosis might be also important reasons for the abdominal apoplexy. Of course the mismatching could also be caused by the high proportion of cases without identifiable bleeding sites and the respective rupture risks of different aneurysms. Arteriosclerosis also was one of the reasons for the aneurysm, especially dissecting aneurysms. It was generally believed that the most common cause of abdominal apoplexy in older patients was arteriosclerosis with or without hypertension. In younger patients congenital defects or systemic disease might account for the hemorrhage such as polyarteritis nodosa and Ehlers-Danlos syndrome.^[1,12,13]^

There were 2 arteries involved in our case: GDA and IPDA. To our knowledge, this is the first reported case of abdominal apoplexy that has 2 identified bleeding sites. Historically, about 70% cases of abdominal apoplexy had identifiable source of bleeding, and there was only 1 bleeding site in each identifiable case. Both GDA and IPDA were the rare sources of hemorrhage. Kennedy reported 2 cases of GDA and 1 case of IPDA in 50 cases of abdominal apoplexy (19 cases undetermined).^[6]^ Carter reviewed 2 cases of GDA and 2 cases of IPDA in 85 cases of abdominal apoplexy (35 cases undetermined). Kleinsasser reviewed 3 cases of GDA and 3 cases of IPDA in 83 cases of abdominal apoplexy (23 cases undetermined). The rupture of GDA usually was caused by artery dissections, but GDA dissections were particularly rare.^[14–16]^ The anatomic area of GDA may be susceptible to mechanical stress contributing to small artery dissection in combination with other poorly defined predisposing conditions.^[16]^ The aneurysm of IPDA was also rare and usually caused by atherosclerosis, pancreatitis, and trauma with the symptoms of gastrointestinal or retroperitoneal hemorrhage.^[17–21]^

The abdominal apoplexy with 2 bleeding arteries was extremely rare, considering the low incidence of each artery in the literature. However, cerebrovascular apoplexy or atraumatic multifocal intracerebral hemorrhage was also extremely rare but had been described in the literature. Multiple contributing factors have been correlated to multifocal intracerebral hemorrhage in the literature, which included hypertension, multiple microbleeding, cerebral angiopathy (such as cerebral amyloid angiopathy), vasculitis, and so on.^[22]^ We believed that there might be some equivalence between multifocal cerebrovascular apoplexy (intracerebral hemorrhage) and multifocal abdominal apoplexy. Without the pathological evidence, the exact etiology of our case was undetermined. But the angiography revealed the absence of obvious aneurysm and the bleeding sites located at the branches of 2 arteries. Considering the history of hypertension and cerebrovascular disease, the possible mechanism in our case might be atherosclerosis, hypertension or multiple microaneurysms. Also, 1 of the 2 arteries might be the primary site of bleeding, and the other was secondary to the traction of retroperitoneal hematoma caused by the primary bleeding.

In the emergency situation of sudden abdominal pain and undetermined hypotension, the awareness of abdominal apoplexy was still the key point in the management of this unexpected catastrophic attack. Diagnostic evaluation specific to abdominal apoplexy was largely dependent on imaging techniques. CT angiogram was very useful in making the diagnosis, localizing the bleeding vessel, and guiding subsequent surgical and angiographic management.^[10]^ In our case, the enhanced CT revealed extravasation of the contrast medium and the diagnosis of abdominal apoplexy was confirmed. Then the subsequent angiography confirmed the bleeding sites and successful transcatheter embolization was performed with satisfied prognosis. Spontaneous stop of bleeding from GDA or IPDA had been described in the literature,^[15,23]^ but this was extremely rare, so the active treatment was widely recommended.

Historically, the nonsurgical mortality had approached 100% and laparotomy was recommended as the first therapy for the abdominal apoplexy. However, the development of imaging technology gave us more opportunities to diagnose the abdominal apoplexy and localize the suspected bleeding vessels, which might somewhat change the treatment strategy of abdominal apoplexy in selected patients. Now embolization was much preferred because of less invasion if the patient was hemodynamically stable.^[1,4]^ But if the patient was hemodynamically unstable with undetermined diagnosis from the CT angiogram, exploratory surgery should be employed.^[10]^ Unfortunately nearly 30% to 40% surgeries could not localize the site of bleeding or just find hematoma of mesentery even through the laparotomy, and reported mortality with nontherapeutic exploratory laparotomy varies from 40% to 66%.^[1]^ The reason for nontherapeutic exploratory laparotomy might include the lower pressure and the site of bleeding. The rupture of GDA or IPDA usually caused hematoma in the retro peritoneum, around the head of pancreas and duodenum. Surgical exploration of this area might be difficult owing to the multiple small vessels from the pancreaticoduodenal arcade and the unexpected risk of damage to the pancreas and duodenum. In the condition of our case, embolism might be a better choice than laparotomy.

## 4 Conclusion

Abdominal apoplexy is a rare and fatal emergency event. The exact mechanism of abdominal apoplexy was unclear, but arteriosclerosis, hypertension, abdominal aneurysm, and other predisposing angiopathy were considered to be the main reasons of abdominal apoplexy. The development of the imaging technology gave us more opportunities to suspect or confirm the diagnosis of abdominal apoplexy. The awareness of abdominal apoplexy was still the key point in the management of this disease. Quick diagnosis by the imaging and immediate embolization were very important for the treatment.
